# The *pK56M* ICAM1 HFpEF risk variant and inflammatory biomarkers

**DOI:** 10.1016/j.ahjo.2023.100346

**Published:** 2023-11-11

**Authors:** Pedro Giro, Kent D. Taylor, Sanjiv J. Shah, Ravi B. Patel

**Affiliations:** a Division of Cardiology, Northwestern University Feinberg School of Medicine, Chicago, IL, United States of America; b The Institute for Translational Genomics and Population Sciences, Department of Pediatrics, The Lundquist Institute for Biomedical Innovation at Harbor-UCLA Medical Center, Torrance, CA, United States of America

**Keywords:** Heart failure with preserved ejection fraction, ICAM-1, hsCRP

## Abstract

**Introduction:**

The *ICAM1* variant rs5491 (*p.K56M*) is common among Black individuals and has been associated with risk of heart failure with preserved ejection fraction (HFpEF). The pathways by which rs5491 leads to HFpEF are not known.

**Methods:**

Among Black individuals within the Multi-Ethnic Study of Atherosclerosis, we evaluated associations of rs5491 with 3 inflammatory biomarkers (high-sensitivity C-reactive protein [hsCRP], interleukin-6 [IL-6], and tumor necrosis factor-α receptor 1 [TNFR-1]).

**Results:**

Among 1558 Black participants (mean age 62 ± 10 y, 47 % female), each additional rs5491 allele was associated with higher hsCRP after covariate adjustment (β: 0.15, SE: 0.07, *P* = 0.02). Each additional rs5491 allele was associated with higher TNFR-1 (β: 0.06, SE: 0.02, P = 0.02), but not IL-6 (β: 0.04, SE: 0.04, *P* = 0.29). The association between rs5491 and HFpEF remained significant after adjustment for hsCRP.

**Conclusion:**

In Black individuals, rs5491 (*p.K56M*) is associated with higher hsCRP and higher TNFR-1, but not IL-6.

## 1. Introduction

Heart failure (HF) with preserved ejection fraction (HFpEF) may be driven by chronic, systemic inflammation due to cardiometabolic comorbidities [1]. Intercellular adhesion molecule-1 (ICAM-1) is a cell surface protein that promotes inflammation through leukocyte transmigration and may play a role in HFpEF. Recently, a common missense genetic variant in *ICAM1* among Black individuals (rs5491; p.Lys56Met) has been associated with increased risk of HFpEF [2]. The pathways by which rs5491 leads to HFpEF are not known. Given the role of ICAM-1, we hypothesized that rs5491 is associated with biomarkers of inflammation. High-sensitivity C-reactive protein (hsCRP), interleukin-6 (IL-6), and tumor necrosis factor-α receptor 1 (TNFR-1) are biomarkers of inflammation, and higher levels of each biomarker are associated with HFpEF [3–5]. We evaluated the associations of rs5491 with these biomarkers in the Multi-Ethnic Study of Atherosclerosis (MESA).

## 2. Methods

Details of the MESA study have been previously described [2]. Data are publicly available at the BioLINCC coordinating center (https://biolincc.nhlbi.nih.gov/home). MESA study personnel recruited community-dwelling adults aged 45 to 84 years from 4 self-identified race/ethnic groups (Black, White, Hispanic, and Chinese) across 6 US field centers. IRB approval was obtained at each site and all participants provided informed consent. Participants were free of cardiovascular disease at Exam 1. Genotyping in MESA was performed using the Affymetrix Genome-Wide SNP Array 6.0 (Thermo Fisher Scientific, Wal-tham, MA); rs5491 was imputed [2]. Inflammatory biomarkers were measured at Exam 1. A BNII nephelometer using a particle-enhanced immunonephelometric assay (N High Sensitivity CRP, Dade Behring Inc) measured hsCRP. IL-6 was measured by an ultra-sensitive sandwich ELISA (Quantikine HS Human IL-6 Immunoassay; R&D Systems, Minneapolis, Minnesota). TNFR-1 was measured using an ultrasensitive ELISA (R&D Systems, Minneapolis, Minnesota). Incident HFpEF was adjudicated by physicians and required both signs and symptoms of HF along with documented ejection fraction >45 % at the time of HF hospitalization [2]. Due to the low minor allele frequency (MAF) of rs5491 in other race/ethnic groups (<5 %), only Black participants in MESA were included. For all analyses, additive models were used for rs5491. In regression models, hsCRP, IL-6, and TNFR-1 were log base 2 transformed. Linear regression models were used to evaluate the associations of rs5491 with biomarkers. We evaluated effect modification of gender on the associations of rs5491 and biomarkers using interaction terms. For all models, covariates for adjustment at Exam 1 were age, gender, body mass index, systolic blood pressure, anti-hypertensive medication use, diabetes, smoking status, and ancestry principal components 1–10. In sensitivity analyses evaluating associations of rs5491 with biomarkers, models were additionally adjusted for household income or lipid lowering medication use. Cox proportional hazards models evaluated the association between rs5491 and incident HFpEF. To evaluate whether the association of rs5491 with HFpEF was independent of hsCRP, we added hsCRP in addition to other covariates in a Cox model evaluating rs5491 and HFpEF. In sensitivity analysis evaluating the association of rs5491 with incident HFpEF, we additionally adjusted for healthcare insurance. Two-sided *p* values <0.05 were considered significant. All analyses were performed in R version 4.0.2 (R Foundation for Statistical Computing).

## 3. Results

**Fig. 1.**
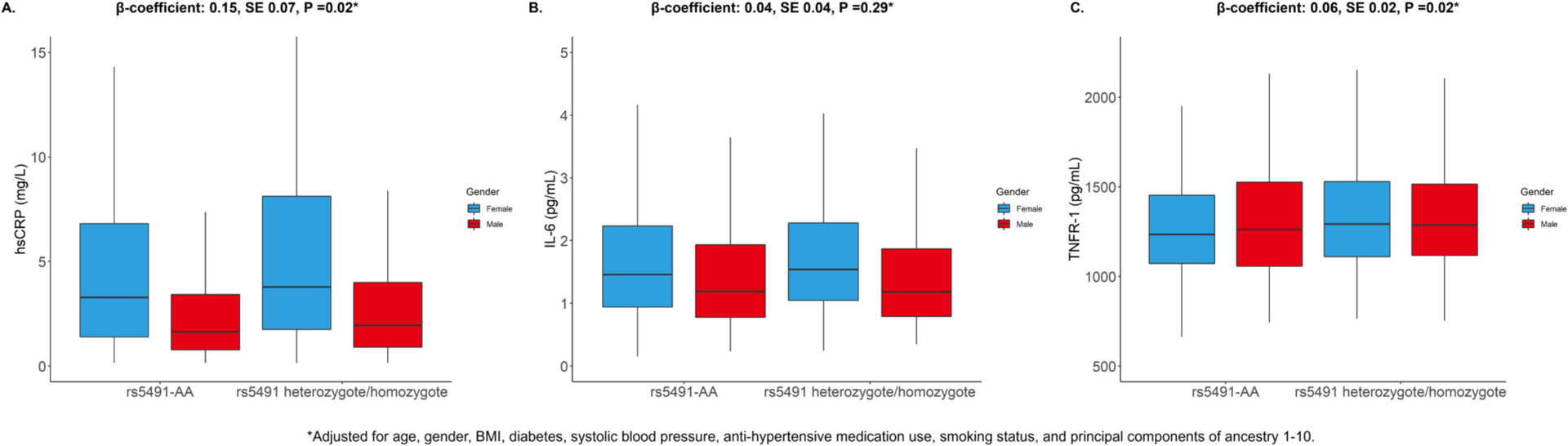
Inflammatory biomarkers at Exam 1 by rs5491 status and gender among Black participants of the MESA study. Box and whisker plots of (A) hsCRP, (B) IL-6, and (C) TNFR-1. Additionally shown above each figure are results of multivariable linear regression models. There was no interaction by gender on the associations of rs5491 with hsCRP (P-interaction = 0.59) or TNFR-1 (P-interaction = 0.63). hsCRP = high-sensitivity C-reactive protein; IL = 6 = interleukin-6; TNFR-1 = tumor necrosis factor-α receptor 1.

Of 6450 individuals in MESA who consented to genotyping and had adequate data availability, there were 1688 Black individuals. Among these 1688 Black individuals, 77 did not have rs5491 genotyping data and 53 were missing covariate data. The final analytic cohort was therefore 1558 participants. Among 1558 Black participants (mean age 62 ± 10 y, 47 % female) with hsCRP available, the MAF of rs5941 was 21 %. After covariate adjustment, each additional rs5491 allele was associated with higher hsCRP (β: 0.15, SE: 0.07, *P* = 0.02; Fig. 1). Of the 1558 participants, there were 634 participants with TNFR-1 levels available for analysis. Each additional rs5491 allele was associated with higher TNFR-1 (β: 0.06, SE: 0.02, *P* = 0.02). Among 1507 individuals with IL-6 available, there was no association between rs5491 and IL-6 (β: 0.04, SE: 0.04, *P* = 0.29). The associations of rs5491 with higher hsCRP and TNFR-1 were consistent by gender (hsCRP P-interaction = 0.59; TNFR-1 P-interaction = 0.53). The association of rs5491 with hsCRP was consistent after further adjustment for income (β: 0.19, SE: 0.07, *P* = 0.01) and use of lipid-lowering medication (β: 0.14, SE: 0.07, *P* = 0.03). Consistency was noted in associations of rs5491 with TNFR-1 after further adjustment for income (β: 0.03, SE: 0.02, *P* = 0.035) and lipid-lowering medication (β: 0.04, SE: 0.02, *P* = 0.02). There were 42 HFpEF events over a median follow-up of 16.5 years. Each additional rs5491 allele was associated with incident HFpEF in the covariate-adjusted model without hsCRP (HR 1.87, 95 % CI: 1.16–3.02, *P* = 0.01), with similar results after further adjustment for hsCRP (HR 1.86, 95 % CI: 1.15–3.01, P = 0.01) and healthcare insurance (HR 1.84, 95 % CI: 1.14–2.96, P = 0.01).

## 4. Discussion

In Black individuals, rs5491 (*p.K56M*) is associated with incident HFpEF, higher hsCRP, and higher TNFR-1, but not IL-6. The association between rs5491 and incident HFpEF persisted after adjustment for hsCRP. Serial hsCRP levels were not available in a sufficient number of participants for analysis and other inflammatory biomarkers, including interleukin-1β, were not available. hsCRP and TNFR-1 are considered biomarkers of inflammation that have been associated with HFpEF, and TNFR-1 may be more sensitive to HFpEF than IL-6 [3,4]. Based on these preliminary findings, further investigations encompassing broader inflammatory biomarkers are required to evaluate the inflammatory effects of rs5491 to better understand the variant’s association with HFpEF.
